# Biomimicry for Regenerative Built Environments: Mapping Design Strategies for Producing Ecosystem Services

**DOI:** 10.3390/biomimetics5020018

**Published:** 2020-05-12

**Authors:** Maibritt Pedersen Zari, Katharina Hecht

**Affiliations:** 1 Wellington School of Architecture, Victoria University of Wellington, Wellington 6012, New Zealand; 2 Department of Biology, Utrecht University, Padualaan 8, 3584 CH Utrecht, The Netherlands; katharina.hecht11@gmail.com

**Keywords:** ecosystem services, biomimicry, urban design, urban ecology, data visualization, sustainability, regenerative design

## Abstract

Built environment professionals must solve urgent and complex problems related to mitigating and adapting to climate change and biodiversity loss. Cities require redesign and retrofit so they can become complex systems that create rather than diminish ecological and societal health. One way to do this is to strategically design buildings and cities to generate and provide ecosystem services. This is an aspect of biomimicry, where whole ecosystems and their functions are emulated, in order to positively shift the ecological performance of buildings and urban settings. A small number of methodologies and frameworks for ecosystem services design have been proposed, but their use is not wide spread. A key barrier is the lack of translational work between ecology concepts and practical examples of ecosystem services design for a built environment context. In response, this paper presents research underpinning the creation of a qualitative relational diagram in an online interactive format that relates ecosystem services concepts to design strategies, concepts, technologies, and case studies in a format for use by built environment professionals. The paper concludes that buildings and whole cities should be expected to become active contributors to socio-ecological systems because, as the diagram shows, many strategies and technologies to enable this already exist.

## 1. Introduction

This paper describes the initial development of an online interactive tool called the ‘strategies for designing urban ecosystem services diagram’. The diagram can be used as a basic tool for architects and built environment professionals to integrate ecosystem services into interdisciplinary built environment design projects, and can be used in conjunction with other tools and methods to work towards holistic regenerative design. The interactive diagram translates ecological concepts into practical examples of built and tested designs. In this way it contributes to closing the gaps between ecological and design knowledge that exist in many design practices. In the field of design, the study of precedents and visualizations of concepts tend to be more commonly utilized educational methods, compared with written scientific explanations of concepts. Visual material tends to be more widely consumed and understood among design professionals. The presented diagram therefore offers a platform that brings scientific ecological knowledge closer to designers with the goal being to shift the ecological effectiveness of biomimetic architectural and urban design from a focus solely on innovation, to one more centered on ecological regeneration. Furthermore, the diagram also potentially allows ecologists to better understand and adapt to the needs of designers and design thinking when working in interdisciplinary contexts. This interdisciplinary collaboration is an important requirement for effective work toward the creation of a regenerative built environment [1]. What makes the online platform and interactive database of ecosystem services focused design methods, strategies, and case studies unique, is that it is accessible as it is being developed, it is interactive, and it is a researched based collaboration between design and ecology/ biology academics. The diagram is interactive and requires users to engage with it online to experience and understand its functionality fully. A link to version 1.0 of the diagram is available by contacting the authors directly. The developed diagram aims to become a complete, accessible, and useful compilation of ecosystem services related design strategies that are valuable for people working in architectural and urban design and planning fields. The paper concludes by describing how in the next stages of the research, the tool will be improved regarding its content, usability, and visualization effectiveness.

### 1.1. Context: The Need for a New Way to Design Built Environments

The way buildings and cities are designed will need to change rapidly to address converging drivers of change such as climate change and biodiversity loss. This must occur within the complex context of human population growth, increased per capita consumption, and global urbanization [2]. Although cities occupy only up to 4% of global land area [3,4], they are the sites of tremendous concentrations of energy use, water use, materials, greenhouse gas (GHG) emissions, and other pollutants. Because of the built environment’s increasing appropriation of the goods and services of ecosystems, vital ecological services for human society (and other species) such as climate regulation, soil formation, nutrient cycling, pollination, and waste assimilation are negatively affected [5]. Modern cities are primarily sites for cultural expression and the facilitation of trade, rather than for the production of physical resources or the generation of ecosystem services that produce tangible physical ecosystem or human health [6]. Urban environments must be considered in terms of their impact on climate and ecosystems, and their potential role in facilitating regeneration of them. 

Changes to the climate, and therefore related impacts on the built environment are expected to increase in intensity in the future. The built environment is responsible for approximately a third of global anthropogenic GHG emissions, leading to climate change [7]. This suggests that a re-evaluation of the built environment and rapid expansion of policies and actions to mitigate greenhouse gas (GHG) emissions are urgently required [8]. The built environment will also have to adapt to climate change impacts, as the main site of human economic, social and cultural life. More than half of all humans now live in urban built environments. It is important therefore that built environment professionals not only work towards mitigating the causes of climate change, but are also able to devise strategies to adapt to climate change impacts. In this regard, many technologies and materials have proven to be important including sustainability focused nature inspired ones [9,10,11,12]. Concurrently, biodiversity loss, and ecosystem degradation must be addressed. The degradation of ecosystems, along with the fact that there is a positively reinforcing feedback loop between biodiversity loss and accelerated climate change [13,14], is why biodiversity loss is an urgent issue to address [15], and why this must be considered in built environment design [16]. Cities and the buildings within them must become ecologically regenerative [17]. 

### 1.2. Regenerative Urban Design

Regenerative design aims to produce quantifiable ecological and social health outcomes rather than to simply minimize energy or water use, or the emission of pollutants [18]. In the context of this article, regenerative design is defined as meaning human made interventions and systems (buildings, urban spaces, infrastructure etc.) that contribute to ecological, social, and cultural health in various holistic and interconnected ways based on definitions provided by Mang and Reed [19] and Hes and du Plessis [20]. The authors acknowledge that humans are an indivisible aspect of multiple interacting ecologies [21]. An important (but certainly not the only) aspect of regenerative design is ensuring that the functioning of the built environment leads to positive outcomes in a biological sense. One way to conceive of this is to devise ways to work towards the provision of regulating, supporting, and provisioning ecosystem services through architectural and urban design [22]. This is the focus of this article, rather than the social and cultural aspects of regenerative design, though these are no less important or necessary. The motivation of the development of the diagram tool described in this paper was to contribute in a small way to wider holistic strategies, methods, and paradigms related to regenerative design.

How then can design professionals practically engage with an ecology-based regenerative design agenda or set of goals? In this regard, there is an obvious and accessible example to investigate and then emulate, namely the living biological world and its complex systems. Ecosystems remain the best-known example of sustainable organization of life on this planet [23]. It is logical therefore to try to understand, and if possible, to emulate how organisms and ecosystems work and what they do in the pursuit of the creation of a regenerative human urban habitat [24].

### 1.3. Biomimicry for Regenerative Built Environments

Looking to the living world, reveals organisms and systems that can be mimicked to create and maintain a resilient and adaptable urban built environment, and improve its capacity for regeneration of the health of ecosystems [22]. This applies to urban scale interventions, through to the scale of building components and materials, and can be termed ‘biomimicry’ [25]. Biomimicry is the emulation of strategies seen in the living world as a basis for design and innovation, and has potential to contribute to the creation of more sustainable architecture and urban environments [26]. It is the emulation of an organism, organism behavior, or an entire ecosystem, in terms of its form, material, construction method, process strategies, or function [22]. Mimicking living organisms or ecosystems involves a process of translation into suitable solutions for the human context. Several noteworthy examples of biomimetic architecture or technologies that can assist the built environment in adapting to climate change or becoming an agent of ecological health are examined by Pedersen Zari [22], Pawlyn [26], Vincent et al. [23] and Vogel [27]. Ecosystem biomimicry, that is the emulation of how whole ecosystems function and the ways in which they work, may be the most effective kind of biomimicry to respond to climate change and biodiversity loss in the context of architectural and urban design [28]. This is because ecosystem biomimicry fits into a paradigm of whole systems thinking and change, rather than the design of single components. Ecosystem biomimicry remains the least explored aspect of biomimicry in built form.

### 1.4. Systemic Improvement of the Built Environment: Ecosystem Biomimicry

Ecosystems are typically resilient and many are able to move through infrequent abrupt changes while still supporting the survival of organisms [29]. The ability of ecosystems to adapt to rapid changes that will come about due to climate change is difficult to predict [30]. Despite this, understanding ecosystems beyond a basic metaphoric level can provide insight into how the built environment could function more like a complex living system rather than as a set of unrelated, object-like buildings, and thus become more resilient and better able to adjust to change.

### 1.5. Mimicking How Ecosystems Work: Process Strategies

Strategies for addressing climate change and ecological degradation that are inspired by a thorough understanding of ecosystem processes (that is, how they work) challenge conventional architectural design and procurement thinking, particularly related to the typical boundaries of a building site and design time scales. By emulating process strategies in ecosystems, designers have successful models to follow in devising how systems in buildings or urban environments should be put together and how they should work. Typically, such systems mimic the processes in ecosystems where waste becomes a resource for another component of the system, or where energy is shared or cascaded, ensuring that the system eliminates or reduces duplication of effort. Well-known examples of industrial ecology such as Denmark’s Kalundborg industrial region demonstrate how the process of cycling materials in ecosystems can be mimicked between diverse companies. In Kalundborg, this sharing of waste as resource results in a reduction of approximately 30 million m^3^ of groundwater used, and a reduction in emission of 154,000 tonnes of CO_2_ and 389 tonnes of mono-nitrogen oxides (NO_x_) [31,32]. The elimination of toxins and pollutants that lead to the degradation of ecosystems is also addressed with such an approach. Examining ecosystem processes other than just the cycling of wastes or sharing of energy, offers several additional strategies for the built environment to mimic. These tend to be less explored in built environment design. 

A group of ecosystem process strategies was formulated in earlier research. These can form the basis of built environment focused biomimetic design related to ecosystem processes [33]. If the emulation of ecosystem processes remains at a metaphorical level however, buildings or neighborhoods that are biomimetic may eventuate, but these may not necessarily be better than conventional designs in terms of ecological performance. 

### 1.6. Mimicking What Ecosystems Do: Ecosystem Services

Analyzing the urban built environment from the perspective of how ecosystems function (that is, what they actually do), and then designing changes to cites, buildings, and building components so that they begin to quantifiably emulate the functions of ecosystems could work towards the creation of cities where positive integration with, and restoration of local ecosystem services could be realized [22,34,35,36]. The ecosystem services framework is one way to understand the complexity of ecosystem functions and human interactions with them. The ‘strategies for designing urban ecosystem services diagram’ has been designed based on the ecosystem services framework presented in Table 1 and uses the same iconography. The research methods and rationalization for this Table are described in the authors’ previous work [22]. Ecosystem services are the benefits that humans (and all living organisms) derive, either directly or indirectly from the functions of ecosystems [37]. Ecosystem services are defined and listed in many different ways [37,38], but typically are divided into: provisioning services such as food and medicines; regulation services such as pollination and climate regulation; supporting services such as soil formation and fixation of solar energy; and cultural services such as artistic inspiration and recreation. A focus on ecosystem services has been widely adopted among ecology and policy professionals [39], and was formalized by the United Nations’ Millennium Ecosystem Assessment of Ecosystems and Human Wellbeing [38].

In urban environments, many ecosystem services are thought to occur at low rates except for cultural ecosystem services [40,41]. Despite this, several important urban ecosystem services have been identified and include: air purification; water flow regulation; micro climate regulation; and carbon sequestration [42]. Typically, these urban ecosystem services come from urban green spaces such as forests and parks, or blue spaces such as lakes, streams, and wetlands. They represent important opportunities for novel design interventions, particularly related to increasing resilience to climate change and increasing human wellbeing [43,44]. Opportunities also exist for green, or grey/green hybrid infrastructure, and for buildings themselves to produce ecosystem services [34,44]. Emulating what ecosystems do (provide ecosystem services) can become the overall ecological performance goal generator for a development, while the specific methods or technologies to achieve the goals can be drawn from a wide range of existing design strategies, concepts, and technologies. These may include specifically biomimetic ones, but non-biomimetic techniques or technologies should not be excluded when seeking to generate ecosystem services through built form. As cities densify, there is less capacity for cities to derive all ecosystem services from urban green and blue spaces [45]. This is a further reason why buildings and infrastructure must begin to produce ecosystem services.

By emulating ecosystem services, a building or development could be designed for example to be part of a system that: produces food; produces renewable energy; produces raw materials for the future built environment; collects and purifies water; purifies air and soil; regulates climate through mitigating GHG emissions and the heat island effect; contributes to soil formation and fertility through careful cycling of bio-degradable wastes and recycling of non-biodegradable wastes; and deliberately and strategically provides habitat for species suitable for co-habitation with humans in urban built environments [22]. New ecologically regenerative developments, meaning in this context built interventions such as buildings, neighborhoods, and infrastructure that increase ecological and social health, could act as filters (mechanisms that purify air and water), producers (of food and materials) and generators (of energy) for the rest of the built environment which is still degrading ecosystems, and is likely to persist for at least another 50 to 90 years [46]. If these regenerative developments start to perform even small aspects of ecosystem functioning, it is possible that if they reach the right scale, some causes of climate change and biodiversity loss attributed to the built environment would be mitigated, and at the same time the built environment could become more adaptable to climate change, while concurrently creating beneficial biodiversity outcomes, and increasing human individual and social health [47].

### 1.7. Ecosystem Services Analysis as a Tool for Regenerative Urban Development 

Ecosystem services analysis (ESA), developed by Pedersen Zari [22], is one means by which the concept of ecosystem services is applied to built environment contexts. The purpose of ESA is to measure past, current and potential future environmental performance of the built environment in terms of ecosystem services provision so that future spatial and temporal ecology derived performance goals can be devised.

**Figure 1:**
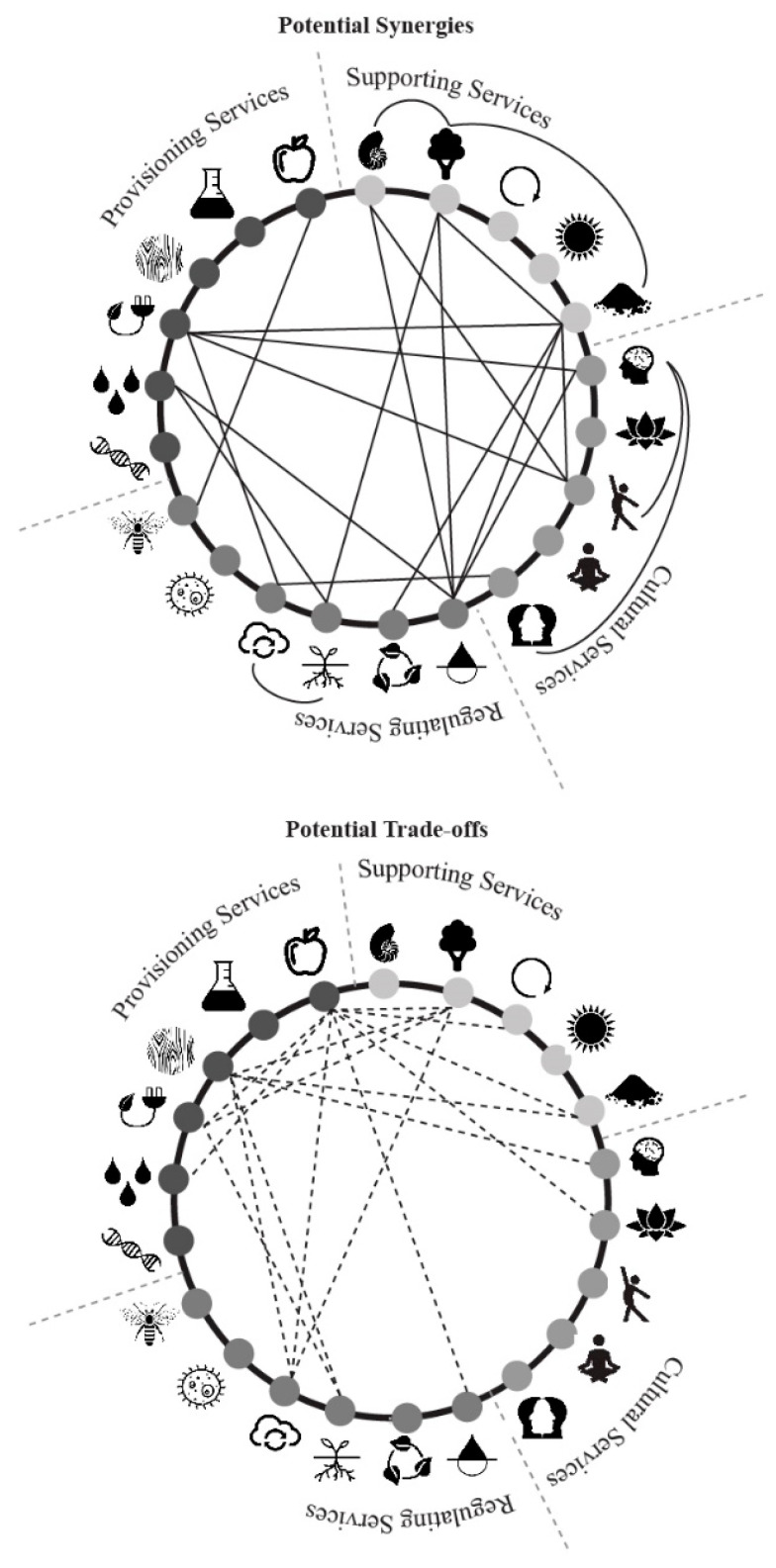
Potential synergies and trade-off relationships between ecosystem services. Source: [49]. See Table 1 for icon key.

If designers and policy makers are to effectively use the ecosystem services model in urban settings, they must understand how these ecosystem services are related however [48]. This is so potential synergies between ecosystem services can be leveraged, but also so that potential trade-off relationships between certain ecosystem services can be avoided or addressed [49]. Recent research by one of the authors [50] illustrates relationships between ecosystem services for a design audience based on quantifiable ecological research results devised by Lee and Lautenbach [51]. Further qualifying ecological and socio-economical associations between ecosystem services were provided by Mouchet et al. [52], Howe et al. [53], Bennett et al. [48], and Raudsepp-Hearne et al. [54] (see Figure 1). Considering the temporal aspects of ecosystem services are also crucial, though often not considered in either the examination of ecosystem services or within a design context [49,55].

The ‘strategies for designing urban ecosystem services diagram’ is both a compilation of existing designs, concepts, and strategies that generate ecosystem services, as well as a relational map that displays existing connections between ecosystem services when integrated in urban designs. It becomes more effective if used in conjunction with an understanding of synergetic or trade-off relationships between existing and potential ecosystem services (as illustrated in Figure 1). For example, a particular project may have a focus on trying to design a building that is able to contribute to purification of air, water, and soil. The ‘strategies for designing urban ecosystem services diagram’ can be used to show that several existing methods such as bioremediation, contaminant absorption, and vertical forests could be investigated for suitability for the project. Case studies demonstrating how such strategies or technologies have been used are also available through the diagram. Figure 1 demonstrates that such a project should also be aware that a singular focus on purification could have synergies with the provision of fresh water, provision of habitat, species maintenance, and soil building ecosystem services if designed strategically. Figure 1 also shows that a potential trade off with purification exists with food provision if not considered at the design stage (this could occur for example if an urban wetland is used for the purification of water in place of an urban orchard).

## 2. Methodology

### 2.1. The ‘Strategies for Designing Urban Ecosystem Services Diagram’: Aims

In order to make the concept of emulating the functioning of ecosystem services in the built environment easier to implement, it is necessary to demonstrate how designers can practically implement ecosystem services focused design at both the architectural and the urban scale. The aims of this research then are twofold: 1/ design and curate a database of existing and developing strategies and technologies that enable the provision of ecosystem services through the medium of buildings, built infrastructure, and cities. 2/ map and illustrate this data so it is in an online interactive format comprehendible for designers or built environment researchers. The intention of the ‘strategies for designing urban ecosystem services diagram’ is that it becomes a tool for designers to use in investigating the practical application of design strategies that have been used to enable built environments to provide ecosystem services, or aspects of them.

### 2.2. Research Process

This research was design-led, rather than based on a set of quantifiable experiments, as is more common in traditional science research. Because of this, this methodology section outlines the steps in the research process. The reasoning behind each step and how these relate to the research aims is discussed concurrently, rather than being separated into discrete sections. 

#### 2.2.1. Step One: Literature and Design Precedent Review

The theoretical framework for the diagram is based on the ecosystem services relationship diagrams and the urban ecosystem services categories described by Pedersen Zari [22]. This work established the initial 22 ecosystem services and 49 subcategories of ecosystem services that were investigated and mapped (see Table 1). A critical literature and design precedent review of existing and developing strategies and technologies that enable the creation of ecosystem services through the medium of building materials and components, whole buildings, built infrastructure, and urban spaces was conducted. This was combined with and compared to international databases of urban nature-based solutions that focus on climate change adaptation or climate change mitigation [56,57,58,59,60]. For every strategy, concept, or technology identified, one or more illustrative built case studies were investigated and summarized. Case studies (114 in total) include: architecture, landscape architecture, urban design, infrastructure design, building technologies/components/materials, and a number of urban development policies.

It should be noted that cultural ecosystem services are benefits that humans obtain from ecosystems related specifically to psychological, cultural, and societal wellbeing. This ecosystem services type has been well investigated in social sciences literature, and socio-ecological models that link cultural services with ecological functions already exist [61]. Due to the existence of such models, the ‘strategies for designing urban ecosystem services diagram’ currently focuses on design strategies, concepts, and technologies for provisioning, regulating, and supporting ecosystem services. Architects and designers are trained to integrate cultural and aesthetic aspects into their design work already and typically are expert at this. This means mapping design strategies that produce cultural ecosystem services is both a large task, but crucially is less urgent in relation to improving the ecological performance of urban environments. Future work is planned that integrates and relates cultural services to existing elements in the ‘strategies for designing urban ecosystem services diagram’.

#### 2.2.2. Step Two: Relational Database Compilation

The results of step one were compiled into a database that identified relationships between design strategies, concepts, and technologies, and specific ecosystem services. Case studies were also added to the database. The database was developed in the Microsoft Excel program (Microsoft Office Professional Plus 2013). Relationships between 160 distinct design strategies, concepts and technologies that work towards ecosystem services generation were defined.

#### 2.2.3. Step Three: Complex System Visualization

The final step was to design an interactive online visualization of the database that captured the ecosystem services and design strategies identified, as well as the relationships between them. In order to create a holistic understanding of complex systems, Suoheimo & Miettinen [62] suggest employing complexity mapping as a tool. A qualitative visualization of complex systems shows interconnections, patterns, and dynamics of the participating elements [63]. In order to understand the relationships between each element in more depth, the complexity map was first drafted in several iterations manually with sticky notes and hand drawn lines on a board. This particular methodology is common in complex systems mapping [62]. 

To ensure effective visualization of the data, several online qualitative complexity mapping web-based software and platforms were tested for suitability. The mapping tools investigated for their usability and functionality included bubbl.us [64], MindMup [65], 7Vortex [66], and Kumu [62]. Kumu was the platform selected due to its flexibility in structuring, connecting, and controlling the visualization of the data. Kumu was created by J. and R. Mohr in 2011 and enables the mapping of relationships and the visualization of complex systems and large datasets. An additional reason for the selection of Kumu was that the developed database spreadsheet (step two of the research process) could be transferred to the platform for automated updating. 

Within the ‘strategies for designing urban ecosystem services’ diagram each discrete ecosystem service, or design strategy, or case study became a small circle within Kumu and is termed an ‘element’. In total, there are 348 elements in the diagram.

**Figure 2:**
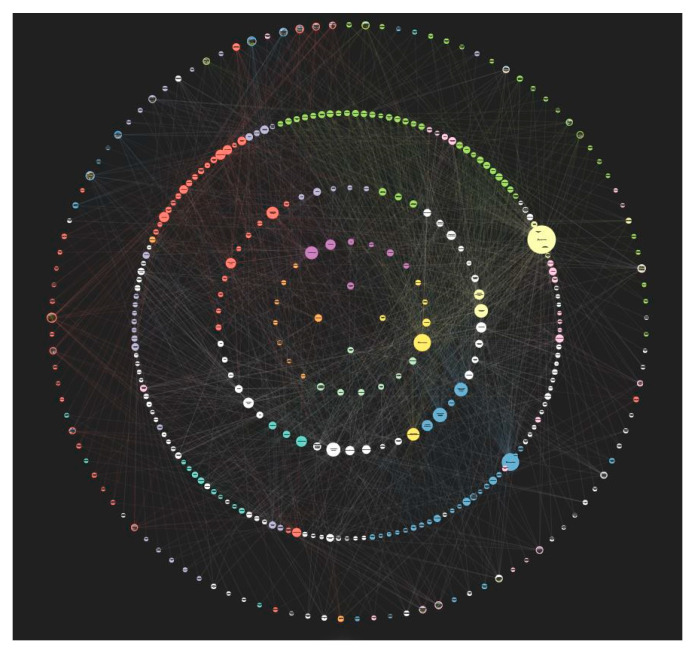
Screenshot of the ‘strategies for designing urban ecosystem services’ diagram version 1.0.

**Figure 3:**
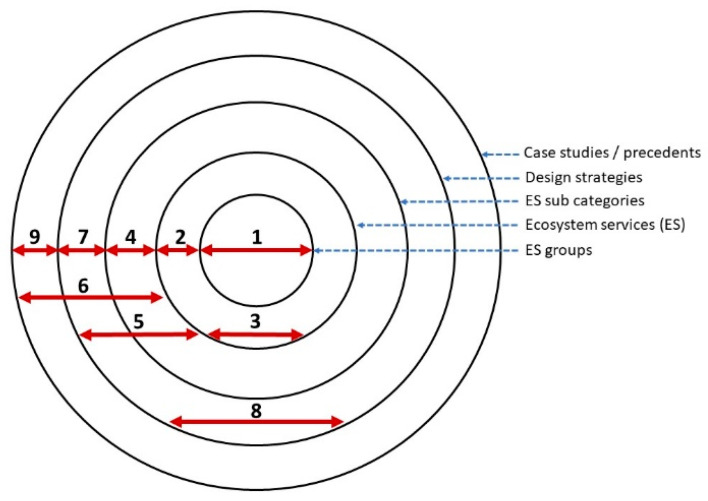
Schematic visualization of inner-circle, or inter-circle relationships line connections (1–9) identified between elements (ecosystem services, subcategories, design strategies, and case studies).

Through an iterative design-led research process, it became clear that organizing the elements into a series of concentric circles made the relational complexity diagram easier to use and understand and meant that users could start from the middle of the diagram with the intention of designing for a specific ecosystem service, or they could start from the outermost circle from the case studies and work their way inwards to understand how multiple ecosystem services can be generated concurrently by understanding a built precedent. The outermost large concentric circle, as seen in Figure 2 is made up of the case studies. The next circle in is the design strategies. The third circle is the ecosystem services subcategories. The second smallest circle is the ecosystem services categories and the innermost circle is the ecosystem services types (provisioning, regulating, and supporting) (Figure 3).

Additional descriptions of each ecosystem service, ecosystem service subcategory, design strategy/technology/concept, and case study were completed and are accessible by clicking on the three grey dots on the left side of the diagram in Kumu. References, links to additional material, and video clips where relevant, were added to the majority of the descriptions. The element colors were determined by affiliation to one of the seven most applicable ecosystem services to a built environment context as defined by Pedersen Zari [22]. These categories are: provision of food; provision of fresh water; provision of fuel/energy; climate regulation; purification; nutrient cycling; and habitat provision. To facilitate the usability of the diagram, icons were used as background images for the ecosystem services categories (see Table 1) and representative photos were used as element images for the case studies. 

**Figure 4:**
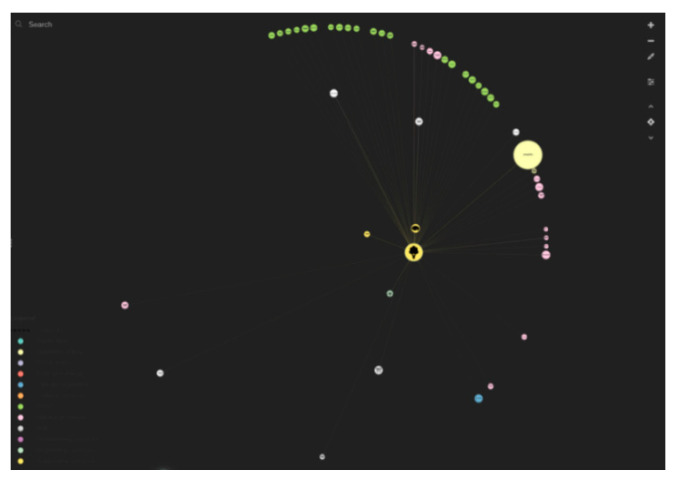
Selecting or hovering over an element highlights the connects to other elements. Screenshot from the ‘strategies for designing urban ecosystem services’ diagram version 1.0.

Each element was manually linked with relationship lines (equating to 1421 individual relationship connections in the diagram). This was done to show how each element is potentially related. For example, if a person selects the element of ‘ecosystem service food provision’ the relationship lines starting from this element become highlighted and show which other ecosystem services, design strategies, and case studies are linked (Figure 4). As people follow these relationship lines, they understand how, practically, to design for the provision of food through the medium of built environment design. Elements are linked between each concentric circle and within them. Figure 3 is a schematic visualization of direct, inner-circle, or inter-circle relationship connections between elements (ecosystem services categories, subcategories, design strategies, and case studies).

## 3. Results

The size of each element in the ‘strategies for designing urban ecosystem services’ diagram was determined through the Kumu program automatically in relation to the amount of direct connections to it. This means the more relationship links an element has, the larger the element circle appears on the diagram. By analyzing the sizes of the elements, it is possible to see which ecosystem services have larger numbers of design strategies attached to them, and which ecosystem services have fewer existing design strategies associated with their creation in a built or urban setting. The diagram displays potential relationships between elements. These relationships can be direct or indirect, which means that certain elements are connected via one or more other elements. For instance, the ecosystem service ‘soil building’ is indirectly related to the ecosystem service ‘decomposition’. This can be explained due to the connection via the ‘soil building’ subcategory ‘renewal of soil fertility’ and the ‘decomposition’ subcategory ‘biodegradation’. 

Analyzing the diagram based on numbers of connections to each element shows that regulating services have the highest amount of connections to design strategies, concepts and technologies (143 connections) while provisioning services have 112 connections. The ecosystem service ‘purification’ showed the highest amount of connections (93 connections) to design strategies, concepts, and technologies among the regulating services, followed by 82 connections to the ecosystem service climate regulation. 

Among provisioning services, most design strategies, concepts and technologies (66 connections) were found for the generation of fuel and energy. The provisioning ecosystem service of fresh water was revealed to have the second most connections to design strategies, concepts and technologies (39 connections). This demonstrates that design strategies, concepts, or technologies that generate provisioning ecosystem services are among the most well-known and developed and are already often integrated into sustainable built environment design. This is not surprising given that these ecosystem services are a familiar and integral part of traditional forms of human economic systems [22,67]. They are tangible, and easily understood.

The type of ecosystem services with the least amount of connections (both direct and indirect) to known design strategies, concepts, and technologies in the diagram was supporting services (109). Supporting services include ecosystem services like ‘soil building’ or ‘nutrient cycling’ and directly support provisioning services. The only exceptions to the low number of known design strategies that relate to supporting ecosystem services were ‘soil building’ which is directly and indirectly related to a total of 84 design strategies, concepts and technologies, and ‘habitat provision’ which reveals 71 connections to design strategies, concepts and technologies. Latter relationships are mostly direct (53 connections) and link to many of the vegetation-related concepts such as living walls, green roofs, community gardens, and urban wildlife corridors etc. Habitat provision was actually the ecosystem service with the highest amount of direct relationships to known ecosystem services design strategies. 

Ecosystem services and sub categories with the least amount of known design strategies associated with them were: provision of genetic information; fixation of solar energy; and control of invasive species. This can be explained because the nature of these ecosystem services relies heavily on communities of living plants, meaning unless plants themselves are integrated into buildings or urban contexts it is difficult for buildings or infrastructure to produce these ecosystem services. Earlier research has shown that if the ecosystem service of habitat provision in urban settings is thought of as a bundle of ecosystem services, including the provision of genetic information, biological control, species maintenance, fixation of solar energy, and soil building, that these ecosystem services can be more readily integrated into urban contexts [22,34].

The ecosystem subcategories of greenhouse gas (GHG) mitigation (38 connections), and climate adaptation (35 connections), which both relate to the regulating ecosystem service of climate regulation, were the largest categories in terms of associated design strategies. This may be a result of current effort in the building and urban design communities to devise strategies for design that addresses climate change. 

Among the design strategies, concepts, and technologies, ‘revegetation’ had the highest amount of connections to ecosystem services. With a total of 98 direct connections ‘revegetation’ relates to many case studies, other design strategies, concepts and technologies, as well as to ecosystem services subcategories and categories. Applying the design strategy ‘revegetation’ in an urban environment can directly or indirectly generate up to 17 different ecosystem services including the provision of habitat, food, fuel and energy, purification and prevention and moderation of disturbances and extremes. This suggests, not unsurprisingly, that the inclusion of green space and living infrastructure into cities and buildings will be an important part of achieving ecosystem services based ecological performance goals in urban settings. Other design strategies with large numbers of connections (meaning they have the potential to contribute to more than one ecosystem service) included urban agriculture and carbon sequestration technologies.

An analysis of the case studies in the diagram and their potential to contribute to urban ecosystem service generation revealed ‘Baubotanik Nagold Tower’ in Germany and ‘Workplace 6’, in Australia were the case studies which produced or contributed to the largest number of ecosystem services. Both have 20 connections to ecosystem services. Therefore, the design strategies or concepts behind these case studies represent potentially effective integrated solutions in the process of creating or evolving regenerative cities in the future. Among the ecosystem services that the case studies contribute to or generate are more common ones, such as the provision of fuel and energy, fresh water, and habitat provision, but also less common ecosystem services, such as biological control and the provision of biochemicals were targeted. 

## 4. Discussion

Examining the, ‘strategies for designing urban ecosystem services’ diagram shows that there are existing design strategies that relate to the emulation, production, or support of every ecosystem service investigated. This suggests that ecosystem biomimicry based on the idea of emulating ecosystem services does not have to rely on new, or un-tested technologies or design ideas. Rather, what is required is an ambitious re-imagining of the overall goals for ecological performance and strategic effort to design buildings or urban spaces that produce multiple interconnected ecosystem services. Provisioning ecosystem services tend to be directly reliant on regulating and supporting ecosystem services [67]. It is important therefore that ecosystem services design does not ignore regulating or supporting services, although these are more difficult to quantify, and indeed to understand and design for [22]. The diagram showed that fewer design strategies were associated with the provision of supporting ecosystem services. This suggests that future research and effort should be made to devise and test design strategies that produce or contribute to supporting ecosystem services more readily. 

That a greater understanding of ecology and systems design is required on the part of design teams is implicit with an ecosystem services approach to architectural and urban design. Increased collaboration between fields that traditionally seldom work together such as architecture or urban design, and biology or ecology would be required. The built environment varies greatly between different climatic, economic, and cultural contexts, and systems that are appropriate to specific places will therefore also vary greatly. Although each differing geographic region will have to evolve its own unique system over time, knowledge of how to create or evolve such systems can be transferred, particularly through ecosystem services design visualization tools such as the ‘strategies for designing urban ecosystem services diagram’.

A whole-system ecosystem services generation approach to built environment design is a suitable solution for a longer-term response to climate change and biodiversity loss, because it addresses many of the underlying issues with current urban environments that are in need of re-evaluation [68]. In this case, issues relate to the fact that the majority of human urban settlements are dependent on fossil fuels to heat, feed, and transport people in a linear system which creates pollution leading in part to climate change. This system also causes the degradation of water ways, air quality, soil, and human health while at the same time consumes non-renewable resources in such a way that they cannot be re-used. A whole-systems pluralistic ecologies approach to built environment design acknowledges that human developments and therefore humans are not in any way separate from the ecosystems they exist in [21].

## 5. Future Work

The ‘strategies for designing urban ecosystem services’ diagram as illustrated in Figure 2 is currently at version 1.0. Version 1.1 will involve verifying the accuracy of existing relationships between diagram elements, and additional expansion of the text descriptions of each element in collaboration with ecologists as appropriate. In order to better indicate and illustrate the nature of existing relationships, connection lines may need to be modified in terms of their direction/strength/color etc. Version 1.1 will also require further collaboration with a graphic designer and software designer to improve graphical and usability issues with the existing Kumu diagram. A revision of how elements are clustered (which currently is determined automatically by Kumu algorithms) will also be useful. Beyond these improvements, it is important to integrate cultural ecosystem services more effectively into the diagram. This is the subject of current ongoing research. The diagram will require continuous development to include new innovations in the area of design for urban ecosystem services. This includes more detailed, scientific, and engineering/architecture focused explanations about ecosystem services and relationships between them, as well as design strategies, concepts, and technologies.

The next major phase of the research will involve testing the developed online system practically in a pilot research project with designers, in order to evaluate the usability of the diagram and to then understand the range of further improvements that should be made. Work on phase 2 in the form of a pilot study using an ecosystem services site measurement tool (ESII Tool 2019 [69]) has been completed with the intention of investigating how existing decision-support tools for ecosystem services measurement [70] can be more effectively used by designers and translated into practical examples of design strategies using the medium of the ‘strategies for designing urban ecosystem services diagram’. This is currently being expanded to include other urban ecosystem services identification and measurement tools suitable for Oceania.

## 6. Conclusions

Mimicking aspects of living organisms can produce innovations that address sustainability issues in some cases, but without an understanding of the ecological context of these organisms, such innovations can too easily become simple technological add-ons or substitution materials in conventional buildings. Such solutions also miss an opportunity to examine the possibility of systemic socio-ecological change in the built environment and to re-evaluate the nature of the relationship between people, their built environment, and the ecologies they exist in.

Positive integration with ecosystems leading to a regenerative rather than damaging effect on them in urban contexts may contribute to maintaining biodiversity and the ecosystem services that humans are dependent upon for survival, particularly as the climate continues to change. Such a concept goes beyond encouraging a basic understanding of ecological processes over time. Instead, it is the thorough integration of quantifiable biological ecological knowledge into architecture and urban design for the purpose of altering how buildings fundamentally function in relation to both ecosystems and to each other. Buildings, and indeed whole cities, should be expected to become active contributors to ecosystems and social systems, rather than remaining unresponsive agents of ecosystem degeneration. 

## Figures and Tables

**Table 1:** Ecosystem Services.

**Supporting Services**	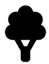	**Habitat provision**	**Cultural services**	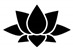	**Aesthetic & artistic inspiration** - Aesthetic value - Artistic inspiration
	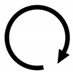	**Nutrient cycling** - Retention of nutrients - Regulation of biogeochemical cycles		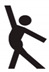	**Recreation and psychological wellbeing** - Sport - Outdoor activities - Tourism - Socialization - Relaxation & psychological benefit
	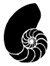	**Species Maintenance**		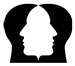	**Sense of place and cultural diversity** - Celebration of cultural diversity/history - Sense of place
	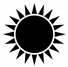	**Fixation of solar energy**		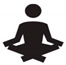	**Spiritual and religious inspiration**
	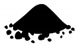	**Soil building** - Soil formation - Renewal of soil fertility - Soil quality control - Soil retention		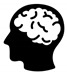	**Education and knowledge** - Educational - Inspiration & innovation - Cognitive development - Knowledge building
**Regulation Services**	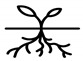	**Disturbance prevention** - Noise - Wave - Erosion - Earthquake - Drought - Flood/Storm events - Wind	**Provisioning Services**	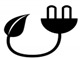	**Provision of fuel and energy** - Water energy - Wind energy - Active/passive solar energy - Human body heat - Hydrogen energy - Biomass energy - Geothermal energy
	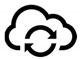	**Climate regulation** - UV protection - Moderation of temperature - Climate adaptation strategies - GHG mitigation		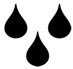	**Provision of fresh water** - Drinking water - Sanitation - Irrigation - Industrial processes - Recreational
	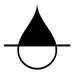	**Purification** - Water purification - Soil purification - Air purification		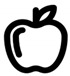	**Provision of food** - Small to large scale urban agriculture
	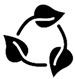	**Decomposition** - Biodegradation - Material reuse/recycling - Consumption reduction		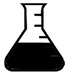	**Biochemicals** - Medicine - Natural chemicals
	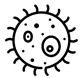	**Biological control** - Control of invasive species - Disease/pest regulation		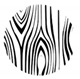	**Raw materials**
	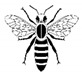	**Pollination**		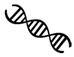	**Genetic resources**
